# Bronchogenic cyst of the ileal mesentery: a case report and a review of literature

**DOI:** 10.1186/1752-1947-4-313

**Published:** 2010-09-23

**Authors:** Adolfo Petrina, Carlo Boselli, Roberto Cirocchi, Piero Covarelli, Emilio Eugeni, Marco Badolato, Luigi Finocchi, Stefano Trastulli, Giuseppe Noya

**Affiliations:** 1 General and Oncological Surgery Unit, University of Perugia, Perugia, Italy; 2 Emergency and General Surgery Unit, University of Perugia, Terni, Italy

## Abstract

**Introduction:**

Bronchogenic cyst is a rare clinical entity that occurs due to abnormal development of the foregut; the majority of bronchogenic cysts have been described in the mediastinum and they are rarely found in an extrathoracic location.

**Case presentation:**

We describe the case of an intra-abdominal bronchogenic cyst of the mesentery, incidentally discovered during an emergency laparotomy for a perforated gastric ulcer in a 33-year-old Caucasian man.

**Conclusions:**

Bronchogenic cyst should be considered in the differential diagnosis of subdiaphragmatic masses, even in an intraperitoneal location.

## Introduction

The laryngotracheal groove appears at the end of the third week of gestation in the embryonic foregut [1]; the dorsal portion of the foregut elongates to form the esophagus, and the ventral portion ultimately differentiates into the respiratory tract, with ciliated epithelium lining both the fetal esophagus and trachea [1-3]. Bronchogenic cyst and esophageal duplications are clinical malformations due to abnormal development of the foregut.

Bronchogenic cysts form from accessory ventral buds arising from the foregut distal to the future lung at about the fifth week of intra-uterine life; the majority of bronchogenic cysts have been described in the mediastinum (90%, most commonly in the posterior aspect of the superior mediastinum [4-8]) and they are rarely found in an extrathoracic location; a small number of them have been reported in abdominal location, with prevalence in the retroperitoneal space [9-12].

We report a bronchogenic cyst incidentally discovered as a small intra-peritoneal mass in our patient, who was admitted to our surgical unit for acute abdominal pain due to gastric ulcer perforation.

## Case report

Our patient, a 33-year-old Caucasian man, was referred to our institution for acute abdominal pain; the symptoms had begun two days earlier as a mild epigastric pain that localized the following day in the right iliac fossa. He had no instances of nausea or vomiting at admission, a body temperature of 37.2°C, a white blood cell count of 20.30 cells/mm^3 ^(polymorphonuclear leukocytes 84.6%) and sluggish peristalsis. He had a history of misuse of a non-steroidal anti-inflammatory drug (NSAID) used to manage his back pain without any medical prescription.

**Figure 1:**
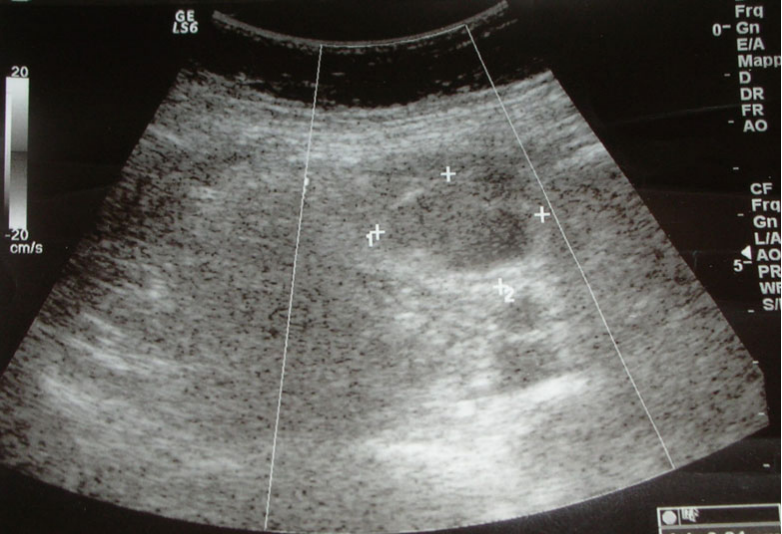
**Ultrasonography showing a 3.2 cm pre-aortic mass**.

Plain X-rays of his abdomen did not show pneumoperitoneum or fluid levels; plain X-rays of his chest were also normal. An abdominal ultrasound scan showed a 3.2 cm pre-aortic mass and some fluid in the Douglas pouch (Figure 1).

**Figure 2:**
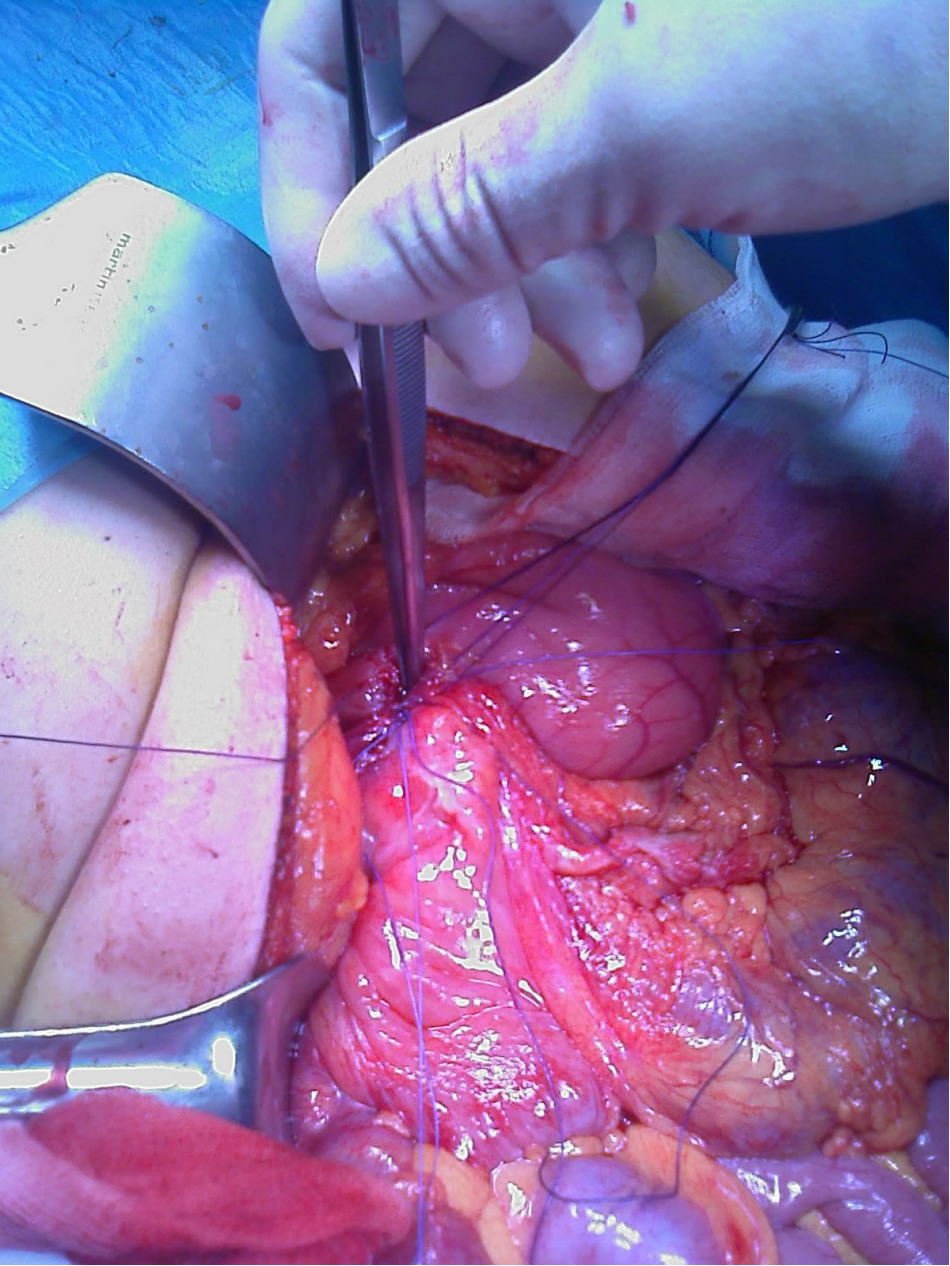
**Perforated gastric ulcer**.

**Figure 3:**
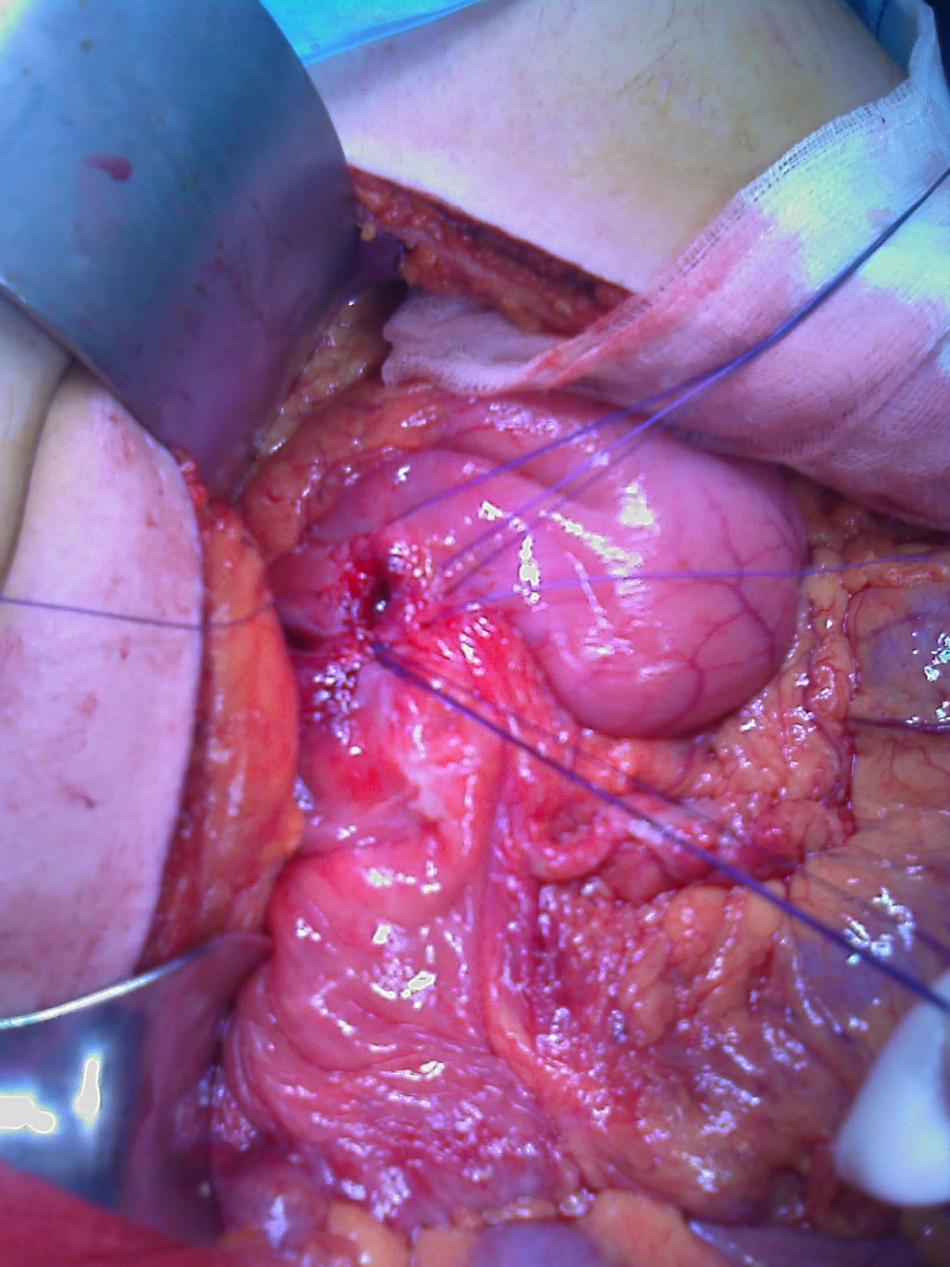
**Perforated gastric ulcer before suture ligation**.

Our patient underwent a laparotomy, which revealed some purulent fluid with mild inflammation of the appendix; the jejunum and ileus were normal. An exploration of the supramesocolic space revealed a gastric perforation of the anterior wall just before the duodenum (Figures 2 and 3).

**Figure 4:**
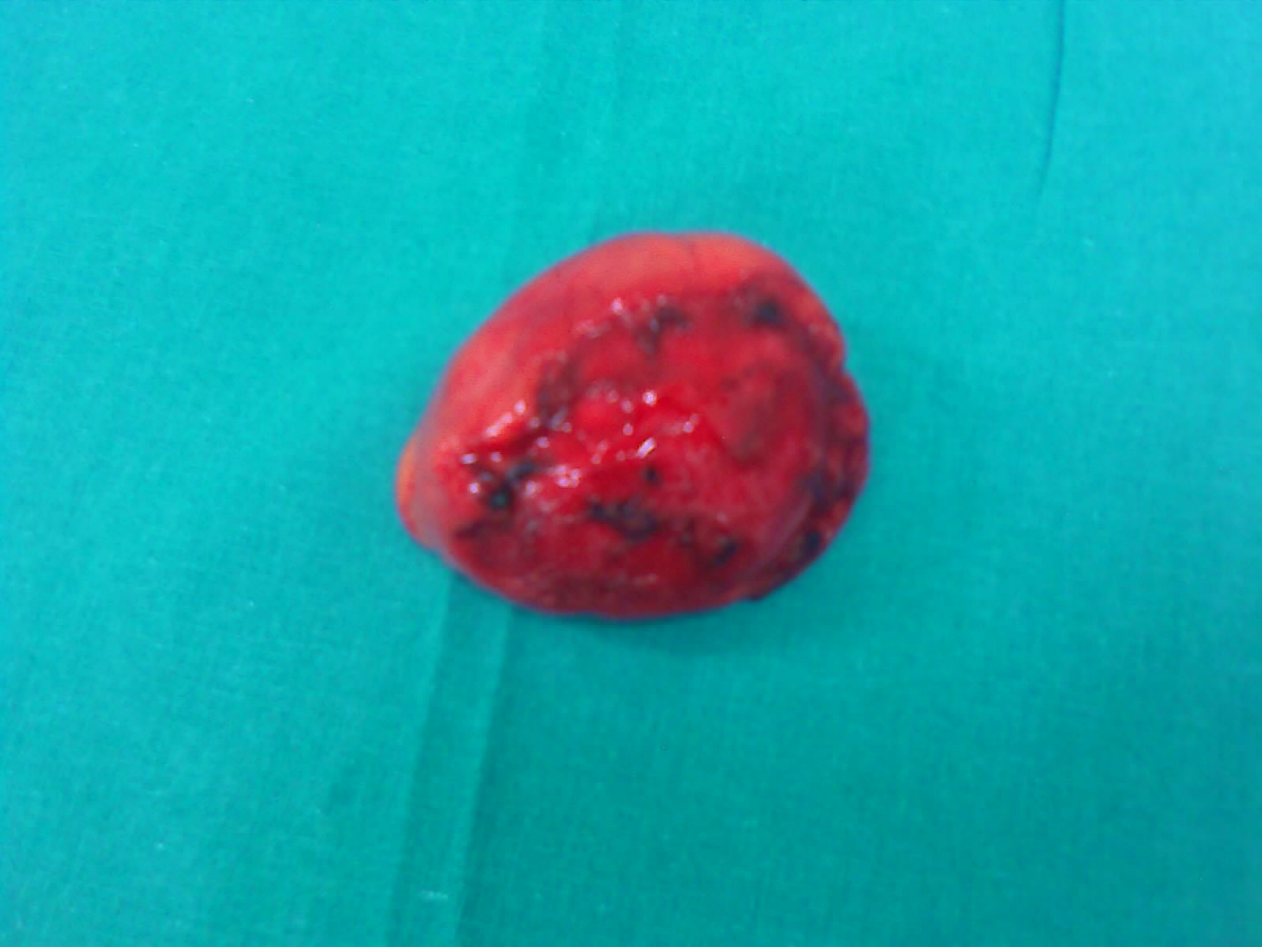
**Ileal mesentery mass revealed on histological examination to be a bronchogenic cyst**.

**Figure 5:**
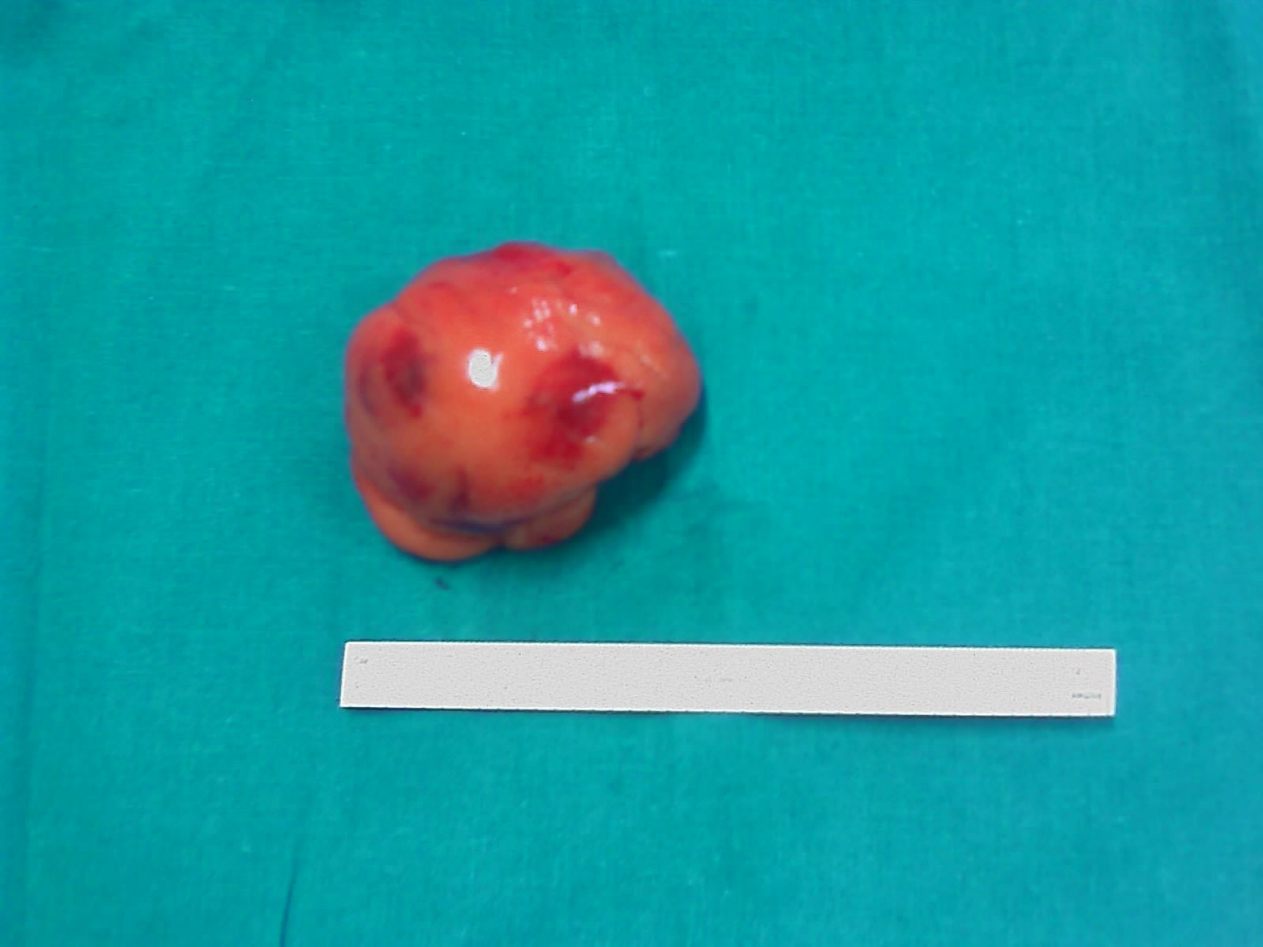
**Ileal mesentery mass (5 cm) revealed on histological examination to be a bronchogenic cyst**.

An appendectomy and suture ligation of the gastric ulcer was performed. Arising from the ileal mesentery was a 5 cm spherical brown mass that on histological examination was revealed to be a bronchogenic cyst (a cyst lined with pseudostratified columnar and ciliated cuboidal epithelium, with a wall of smooth muscle bundles and mucinous glands) (Figures 4 and 5).

Our patient was discharged on the twelfth post-operative day.

## Discussion

Bronchogenic cysts originate from an accessory lung bud of the primitive foregut after the third week of embryonic life. Most commonly they migrate caudally with the esophagus and are eventually found in the posterior mediastinum near the carina, attached to the tracheobronchial tree or to the esophagus. Rarely the cyst may separate completely from its origin and may be found in unusual sites, such as pericardium, skin [13,14] or in intra-spinal locations. Most bronchogenic cysts are small and are usually discovered incidentally because patients are asymptomatic, though sometimes there can be epigastric or left upper quadrant abdominal pain. Malignant transformation is rare [15].

A subdiaphragmatic location is extremely rare, with only about 20 cases reported in the literature [13-19]. This is due to the migration of the cyst prior to the fusion of the pleuroperitoneal membrane. Our patient's cyst was unilocular and arose from the ileal mesenterium, and was filled with mucin.

## Conclusion

Bronchogenic cyst should be considered in the differential diagnosis of subdiaphragmatic masses, even in intra-peritoneal location.

## Consent

Written informed consent was obtained from the patient for publication of this case report and any accompanying images. A copy of the written consent is available for review by the Editor-in-Chief of this journal.

## Competing interests

The authors declare that they have no competing interests.

## Authors' contributions

AP analyzed and interpreted the data from our patient; CB and EE were major contributors to the writing of the manuscript. All authors read and approved the final manuscript
